# Modeling of Flexible Polyurethane Foam Shrinkage for Bra Cup Moulding Process Control

**DOI:** 10.3390/polym10050472

**Published:** 2018-04-26

**Authors:** Long Wu, Kit-Lun Yick, Sunpui Ng, Yue Sun

**Affiliations:** 1 Apparel & Art Design College, Xi’an Polytechnic University, 19 Jinhua South Road, Xi’an 710048, China; wulong7398@163.com; 2 The Institute of Textiles and Clothing, The Hong Kong Polytechnic University, Hung Hom, Kowloon, Hong Kong, China; 16900113r@connect.polyu.hk; 3 Hong Kong Community College, The Hong Kong Polytechnic University, Hung Hom, Kowloon, Hong Kong, China; ccspng@hkcc-polyu.edu.hk

**Keywords:** polyurethane foams, bra cup moulding, process control, thermal conductivity, shrinkage

## Abstract

Nowadays, moulding technology has become a remarkable manufacturing process in the intimate apparel industry. Polyurethane (PU) foam sheets are used to mould three-dimensional (3D) seamless bra cups of various softness and shapes, which eliminate bulky seams and reduce production costs. However, it has been challenging to accurately and effectively control the moulding process and bra cup thickness. In this study, the theoretical mechanism of heat transfer and the thermal conductivity of PU foams are first examined. Experimental studies are carried out to investigate the changes in foam materials at various moulding conditions (viz., temperatures, and lengths of dwell time) in terms of surface morphology and thickness by using electron and optical microscopy. Based on the theoretical and experimental investigations of the thermal conductivity of the foam materials, empirical equations of shrinkage ratio and thermal conduction of foam materials were established. A regression model to predict flexible PU foam shrinkage during the bra cup moulding process was formulated by using the Levenberg-Marquardt method of nonlinear least squares algorithm and verified for accuracy. This study therefore provides an effective approach that optimizes control of the bra cup moulding process and assures the ultimate quality and thickness of moulded foam cups.

## 1. Introduction

Due to the growing importance of the intimate apparel market, there has been an ever-increasing interest in the bra cup moulding process for making seamless bras. In the bra cup moulding process, a pair of hot aluminum male and female moulds (~200 °C) with conformed shapes is used to mould a piece of flat and flexible polyurethane (PU) foam sheet into a specified three-dimensional (3D) cup shape to fit the breast contour of the targeted customer. In common practice, the geometrical data of a specific 3D bra cup are acquired by a laser scanner. The data are further manipulated in a computer-aided design (CAD) system [1] and transferred to a computer numerical control (CNC) machine to mill two aluminum blocks which form a pair of male and female moulds. The specified 3D bra cup shape and volume are different to that of the cavity between the male and female moulds because the flexible PU foam sheet shrinks after moulding and cooling. The CAD operator must determine the foam shrinkage performance and incorporate this information when manipulating the scanned surface data. This is not an easy task because the thickness of the foam cup varies from the least amount of thickness at its edge to the most amount of thickness at its cup tip, i.e., the shrinkage ratio may vary along the cross-sectional geometry of the bra cup. As a result, the mould head design and bra-making process is highly complex, time-consuming, and error-prone due to the large variations in foam properties, cup styles and sizes, and geometric features of graduated padding. Moreover, the PU foam used for bra cup moulding is available in a diversity of hardness, thickness, and density as it is made from different chemical recipes that possess a wide range of physical, thermal, and mechanical properties which affect the degree of foam shrinkage after moulding [2]. To expedite control of the moulding process and to avoid costly trials and errors, previous studies [3,4] have identified the key material properties of PU foam materials that affect the cup shape after moulding, the level of breast support, as well as the tactile feel of the bra cup. The optimal temperature and dwell time for moulding bra cups are greatly affected by the compressive strain and the softening temperatures of PU foams measured by thermo-mechanical analyses. To evaluate the 3D geometrical shape of foam cups, several methods have been proposed to assess the 3D shape conformity and thickness of the moulded cups in objective ways [5,6]. More recently, a new parameterized-based remesh algorithm method has been introduced to measure the 3D shapes of the convex surface of scanned cup samples [7,8]. The shape conformity of the cup was successfully quantified in accordance with the corresponding mould head to determine the optimal moulding conditions, i.e., temperature and duration. Nevertheless, the results indicated that the shape of the foam cup can only adequately fit the aluminum mould head in the region of the cup tip even though the foam cup was moulded in optimal conditions. Substantial variations in cup shape and thickness were consistently observed, and the control of the bra cup moulding process is still far from reliable.

To enhance the quality and the process control of bra cup moulding, the mechanisms of heat transfer and the thermal conductivity of two kinds of PU foams are examined in this study. Dawson et al. [9] developed a model as functions of pressure and temperature using exponential equations to predict the relationship between the thermal conductivity and the specific volume behavior of amorphous polymers. A significant effect of temperature on the thermal conductivity of the material under the conditions of pressure in industrial polymer processing was found. Tseng et al. [10] proposed an analytical model to estimate the thermal conductivity of PU foam via heat transfer mechanisms. Erick et al. [11], and Fellah et al. [12], from a wave propagation aspect, provided different models of transmission of acoustic waves for porous materials with physical parameters. Placido et al. [13], and Wu [14] analyzed different foam insulation morphological structures and established a geometrical cell model which was applied to predict foam insulation properties. In their work, the geometrical structure of cellular foams played an important role in the study of thermal properties. Tao et al. [15] established a model for the thermal conductivity of open-cell, rigid PU foams from the ASTM C 518 method and validated the predicted values which were compared with experiment data. Wang and Pan [16] used a high-efficiency lattice Boltzmann method to numerically study the thermal conductivity of open-cell foam material.

The moulding process of bra cups transfers heat through the PU foam. The moulding temperature, dwell time, and mould gap determine the effects of the thermal conductivity of PU foam. In this work, an analytical model has been developed to address the shrinkage behavior of PU foams based on theoretical and experimental investigations of thermal conductivity. The model will not only provide a better understanding on the shrinkage mechanisms of foam materials, but will also help determine the appropriate moulding conditions for the bra cup moulding process and facilitate better quality control of the ultimate thickness of bra cups.

## 2. Materials and Methods

### 2.1. Materials

In this study, the materials used are flexible PU foams provided by a commercial bra cup manufacturer (Regina Miracle International Ltd., Shenzhen, China). The physical properties of the materials are summarized in Table 1 below. Both of the foam samples (I and II) have open-cell structures which have a relatively high water absorption and air permeability. The size of the sample sheets for the test is 20 cm × 20 cm × 1 cm.

### 2.2. Microscopy Analysis

Due to the importance of the geometric structure of cellular foams as mentioned above, scanning electron microscopy (SEM) with a JEOL JSM-6490 (Tokyo, Japan) microscope was used in the investigation of surface morphology. By examining the SEM images of different foam samples, the reticular or cellular structures can be observed [15]. In the simplest case of the reticular structure of the PU foam, cells are open and the bulk material entirely concentrates on the cell sides, shaping the “struts”. The interstitial gas is the same as the external environment gas, which is typically air. The images observed from SEM were used to determine the pore structure of the foams, including cell shape, cell diameter, cell area, etc.

### 2.3. Moulding of PU Foam Specimens

**Figure 1:**
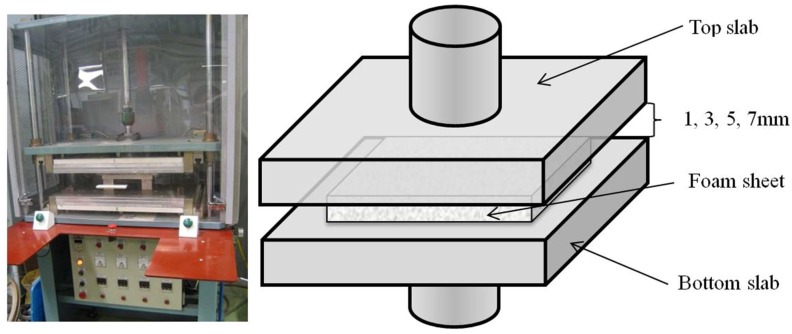
The experimental setup of the moulding machine.

A contour moulding machine, New Pads moulding machine type DM-021HP4-2PR (NPI Co., Taiwan, China), which is commonly used in the industry, was employed to mould the PU foam sheets at different temperatures and durations. In this study, two aluminum top and bottom slabs are installed instead of two mould heads, and foam sheets that are 10 mm in thickness are compressed between hot and parallel slabs at four different distances apart, i.e., 1, 3, 5 and 7 mm. Four pairs of standard rectangular bars were milled in the mentioned thicknesses and placed between the slabs to control the compression strain of the foam specimen during the moulding process. A PU foam sheet was placed on top of the bottom slab while the top slab was vertically brought down by compressed air and automatically lifted up after a duration of pre-set moulding time. Both slabs were heated to a predetermined temperature under the control of thermostats. The experimental setup of the moulding machine is shown in Figure 1. The ultimate thickness of the foam sheet after moulding is affected by the compression strain, the thermal and mechanical properties of the foam, and the moulding conditions. Specimens were allowed to cool down at room temperature for 24 h before the sheet thickness was measured [17]. The foam sheet specimens were moulded in temperatures which ranged from 180 to 210 °C in intervals of 10 °C. The dwell time of the moulding process ranged from 60 to 180 s at 30 s intervals which were controlled by the moulding machine timer (NPI Co., Taiwan, China).

### 2.4. Heat Transfer Mechanisms

Polymers typically have low thermal conductivities, and consequently, the effects of the thermal conductivity of amorphous polymers are key to modeling under steady-state conditions [18]. The thermal conductivity controls the rate of heat transfer within the polymer and is relative to material composition, density, porosity, temperature, etc. [19]. During a high temperature manufacturing process, such as moulding, the polymer is first deformed and heated in a short period of time (e.g., 2 min), and then it is fully relaxed and cooled afterwards. The final size and shape are highly dependent on the material’s thermal conductivity, and it is therefore very important in determining the optimum processing conditions [20,21,22]. The thermal conductivity is the amount of heat transported per unit time through a unit area of a material with a unit length, per degree Kelvin of temperature difference between the two free ends of the material at a steady-state heat flow. For a given polymeric structure, the morphology, formulation, humidity, temperature, and pressure are the most important factors which affect the thermal conductivity [23].

Heat is transferred through the PU foam via four mechanisms: (1) gas conduction through the foam cells, $K_{gas}$; (2) solid conduction through the PU struts, $K_{cond}$; (3) radiation from the foam surface, $K_{rad}$; and (4) convection of gas inside the foam cells, $K_{conv}$. The total heat flow is a result of the interactions between the four modes below [10]:

(1) $$K_{eff}=K_{gas}+K_{cond}+K_{rad}+K_{conv}$$

The thermal conductivity due to conduction in the cell gas mixture has been calculated by using a modification of Wassiljewa’s equation [24] as shown below:

(2) $$K_{gas}=\sum_{i=1}^{n}\frac{y_{i}\cdot k_{i}}{\sum_{j=1}^{n}y_{j}\cdot A_{ij}}$$

where $K_{gas}$ is the thermal conductivity of the gas mixtures, $k_{i}$ is the thermal conductivity of the pure component *i*, and $y_{i}$ and $y_{j}$ are the molar fractions of the components *i* and *j*, respectively. $A_{ij}$ is suggested by Mason and Saxena in the following form [10]:

$$A_{ij}=\frac{\varepsilon {\left[1+{\left(\frac{k_{tr,i}}{k_{tr,j}}\right)}^{\frac{1}{2}}\cdot {\left(\frac{M_{i}}{M_{j}}\right)}^{\frac{1}{4}}\right]}^{2}}{{\left[8\left(1+\frac{M_{i}}{M_{j}}\right)\right]}^{\frac{1}{2}}}$$

where $M_{i}$ and $M_{j}$ are the molecular weights of the pure components i and j (g/mol), $k_{tr,i}$ and $k_{tr,j}$ are the monatomic values of the thermal conductivity of the pure components i and j, and ε is an empirical constant.

Solid conduction in PU foams takes place through the cell walls and struts. The thermal conductivity due to the contribution of conduction through the polymer can be described by:

(3) $$K_{cond}=\frac{2}{3}K_{\rho }(1-\frac{f_{s}}{2})\frac{\rho_{f}}{\rho_{s}}$$

where $K_{\rho }$ is the thermal conductivity of the solid polymer (W/mK). The thermal conductivity of the solid PU polymer from crushed foam samples is reported to be 0.25 W/mK [25] and the fraction of solids in the struts, $f_{s}$, has been reported to be 0.8 [26]. $\rho_{f}$ is the density of the foam (kg/m^3^), and $\rho_{s}$ is the density of the solid polymer (kg/m^3^).

The radiation model of heat transfer refers to the transport of energy by electromagnetic waves, and the attenuation of radiation takes place in the forms of reflection, absorption, and scattering. The radiation between the cell walls has been calculated by the Rossland equation [27] with the extinction coefficient of the cell wall material suggested by Glicksman [26].

(4) $$K_{rad}=\frac{16\sigma T^{3}}{3K},\;\mathrm{where}\;K=\frac{4.1}{d\sqrt{\frac{f_{s}\rho_{f}}{\rho_{s}}}}$$

where σ represents the Stephan-Boltzman constant (5.67 × 10^−8^ W/m^2^K^4^), T represents the Kelvin temperature (K), K represents the extinction coefficient (m^−1^), and *d* represents the cell diameter (m) [16].

The remaining mode of heat transfer is natural convection within the pores. It is negligible since the pore size is so small (<0.5 mm) that the Rayleigh number is much less than the critical value (~1000) for the onset of convection [10,28].

### 2.5. Thickness Measurement

In industrial practice, the thickness of a moulded bra cup is measured from its cross-section which is obtained by cutting the cup through its peak. This destroys the cup sample and involves handling errors [29]. In this study, a noncontact optical microscopy measuring instrument (LEICA M165C, Wetzlar, Germany) is adopted to scan the foam sheet and confirm its thickness before and after moulding [30,31]. The thickness of the foam samples taken at three points which are evenly distributed in the transverse direction was accurately identified, and there were three replicates for each group.

### 2.6. Shrinkage Ratio

Both industrial and experimental studies have revealed that different moulding temperatures and dwell times could result in thermal shrinkage, in which the foam cup thickness is generally smaller than the gap distance between the male and female mould heads. In this study, the shrinkage ratio of the foam sheets is described below [32].

(5) $$SR=\frac{T_{t}-T_{0}}{T_{Original}-T_{0}}+0.1$$

where SR is the shrinkage ratio; $T_{t}$ is the postmoulding thickness of different dwell times (mm); $T_{0}$ is the thickness of the longest dwell time (mm); and $T_{Original}$ is the pre-moulding foam thickness (mm).

## 3. Results and Discussion

### 3.1. Scanning Electron Microscopy (SEM)

**Figure 2:**
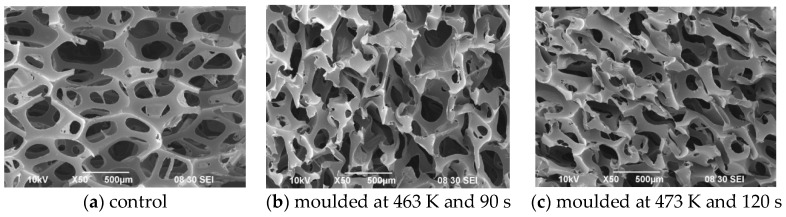
Cellular structure of PU foam (Foam I) under different moulding conditions.

The cellular structures of Foam I under different moulding conditions are presented in Figure 2. A microscopic view of the pore structure of Foam I is shown in Figure 2a. The solid matrix is made of struts, and the cell shape resembles a pentagonal dodecahedron [13]. Moreover, strut thickness is always much smaller than cell diameter. The cells are randomly oriented, and they are mostly homogeneous in size and shape [30]. The pore size varies from approximately 0.4 to 0.8 mm. The mean value of the cell diameter which characterizes the cellular structure is 0.543 mm. Figure 2b,c show images of the same foam that has been moulded under different conditions. It is clear that the cell struts are deformed after compression at a moulding temperature of 463 K and dwell time of 90 s, while the pore walls and struts are further distorted under the moulding condition of 473 K and 120 s.

### 3.2. Shrinkage Ratio and Dwell Time

**Figure 3:**
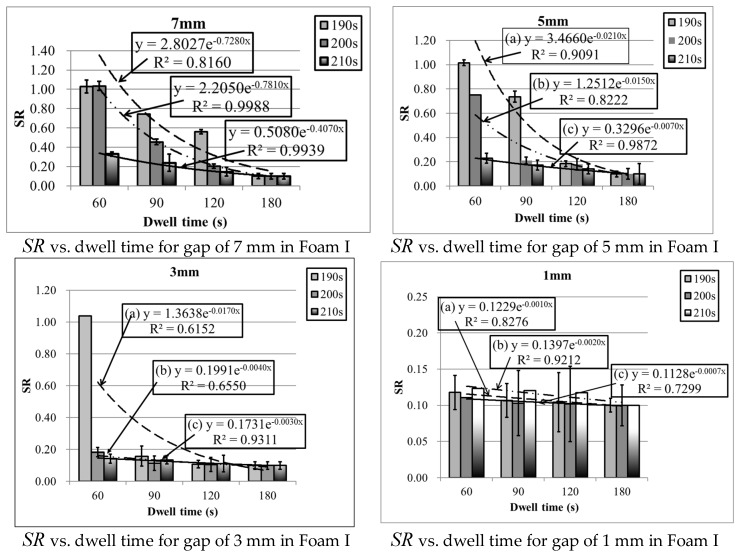
Regression curves of dwell time vs. moulding temperature with different gaps.

The changes in foam shrinkage ratios with different lengths of dwell time are presented in Figure 3. As shown, the shrinkage ratio appears to exponentially decline with the dwell time except at the state where it fails to reach the threshold for forming foam. At lower temperatures and shorter dwell times, the shrinkage ratio takes on a greater value and vice versa. Through a nonlinear regression model, which is an exponential equation, the shrinkage ratio can be predicted by dwell time as well as two empirical constants, *A* and *B*, with reasonable accuracy.

(6) $$SR=Ae^{-Bt}$$

where SR means shrinkage ratio, and t means dwell time (s).

With reference to the Arrhenius equation [33], pre-exponential factor *A* and empirical constant B control the variation trend of the shrinkage ratio. As shown in Figure 3, the PU foam has a higher shrinkage ratio at a relatively low temperature. With the extension of dwell time, the shrinkage ratio rapidly drops. If the dwell time continues to be further extended, the decline rate of the shrinkage ratio will be reduced and a balance is obtained.

### 3.3. Thermal Conduction Analysis

Since the ultimate thickness of the bra cup will also be affected by the moulding temperature, the gap distance between the male and female mould heads and the foam material properties during the thermal conduction process for bra cup moulding is investigated. It is necessary to find the relationship among the three influential factors of conductive and radiative properties with the two coefficients of the exponential equation.

(7) A=f(Gap,Temp,Foam properties)

(8) B=f(Gap,Temp,Foam properties)

As previously shown in the equations, the dependence of radiative and conductive properties are relevant to the moulding temperature, the dwell time, and the geometrical parameters which characterize the internal foam structure, such as the mean wall thickness, the mean strut, and the cell diameters [13]. As the density of foam varies with the gap between the two slabs, the equation can be deduced as shown below.

(9) $$K_{cond}=\frac{2}{3}K_{\rho }(1-\frac{f_{s}}{2})\frac{\rho_{f}}{\rho_{s}}=\frac{2}{3}\cdot 0.25\cdot (1-\frac{0.8}{2})\cdot \frac{1}{1100}\cdot \frac{T_{0}\cdot \rho_{0}}{T_{h}}=f(T_{h})$$

Foam I: $K_{cond}=\frac{0.001}{T_{h}}$ and Foam II: $K_{cond}=\frac{0.00068}{T_{h}}$ where $T_{h}$ means the gap between the two slabs (m).

(10) $$K_{rad}=\frac{16\sigma T^{3}}{3\frac{4.1}{d\sqrt{\frac{f_{s}\rho_{f}}{\rho_{s}}}}}=\frac{16\cdot 5.67\cdot 10^{-8}\cdot T^{3}\cdot T_{h}\cdot d_{0}\cdot \sqrt{1100\cdot T_{h}}}{3\cdot 4.1\cdot 0.01\cdot \sqrt{0.8\cdot \rho_{0}\cdot 0.01}}=f(T,T_{h},d_{0})$$

where $\rho_{f}=\frac{0.01\cdot \rho_{0}}{T_{h}}$ and $d=\frac{T_{h}}{T_{h0}}\cdot d_{0}$.

As the cells are open and the interstitial gas is the same as the external environment gas as previously described, then $K_{gas}$ could be derived from Equation (2) below:

(11) $$K_{gas}=0.001\cdot (0.0793\cdot T-6.99)=f(T)$$

where $K_{gas}$ is the thermal conductivity due to the contribution of conduction through the gas in the cell and T is the temperature in K. Table 2 shows the thermal conduction values under different conditions of Foams I and II.

As evidenced from Table 2, the conduction in the cell gas mixture stands for the main part of the thermal conductivity of foam. About 65–80% of the insulation capacity of a foam is due to the cell gas mixture, while cell size and density contribute to the remaining portion [34]. Due to a smaller variation range of moulding temperatures, the heat transfer of $K_{gas}$ essentially remains unchanged compared to the other two factors, which are solid conduction and thermal radiation. Therefore, in regression models, only $K_{cond}$ and $K_{rad}$ are used to predict the effects of solid conduction and radiation on the two coefficients of an exponential equation, and nonlinear regression models, which use the Gauss-Newton algorithm, are adopted for fitting.

### 3.4. Regression Model

The relationships between the coefficients of the exponential equation and the two heat transfer modes, which are $K_{cond}$ and $K_{rad}$, are examined. A software of Table Curve 3D (SYSTAT, San Jose, CA, USA) by nonlinear regression was used. The fitting procedure was based on the Gauss-elimination algorithm by minimizing the sum of the squares of errors between the data and model regressions. A nonlinear regression was then undertaken, and coefficients can be derived as shown in the equation below:

(12) $$A=a_{1}+\frac{b_{1}}{\sqrt{K_{cond}^{3}}}+c_{1}\cdot K_{rad}$$

(13) $$B=a_{2}+\frac{b_{2}}{\sqrt{K_{cond}^{}}}+\frac{c_{2}}{\ln K_{rad}}$$

where A and B are calculated as the two coefficients of the exponential Equation (6); $K_{cond}$ (W/mK) and $K_{rad}$ (W/mK) are the solid and radiative conductivities, respectively; and $a_{1}$, $a_{2}$, $b_{1}$, $b_{2}$, $c_{1}$, and $c_{2}$ are the constants. Through the equations, coefficients A and B could be predicted according to effective thermal conductivity due to solid conduction and radiation. An example of the regression model for Foam I is formulated as shown below:

(14) $$A=-0.0256+\frac{0.0153}{\sqrt{K_{cond}^{3}}}-2299.6913K_{rad}$$

(15) $$B=0.1506+\frac{0.0246}{\sqrt{K_{cond}}}+\frac{1.9564}{\ln K_{rad}}$$

The determination coefficients ($R^{2}$) of regression Equations (14) and (15) obtained from Foam I are higher than 0.89 with a significance level ≤0.001.

Another regression model for Foam II is formulated as shown below:

(16) $$A=-0.0093+\frac{0.0036}{\sqrt{K_{cond}^{3}}}-894.4124\cdot K_{rad}$$

(17) $$B=0.0765+\frac{0.0115}{\sqrt{K_{cond}^{}}}+\frac{1.0360}{\ln K_{rad}}$$

In the same way, the determination coefficients ($R^{2}$) of regression Equations (16) and (17) obtained from Foam II are higher than 0.83, with a significance level ≤0.001.

In accordance with the relationship between the shrinkage ratio and the coefficients of the exponential equation in Equation (6), and combining the dwell time, the whole shrinkage ratio equation is built as shown below:

(18) $$SR=(A_{1}-\frac{A_{2}}{\sqrt{K_{cond}^{3}}}+A_{3}\cdot K_{rad})\cdot e^{\left(A_{4}+\frac{A_{5}}{\sqrt{K_{cond}}}+\frac{A_{6}}{\ln K_{rad}}\right)}+A_{7}$$

From Equation (18) above, there are a total of seven coefficients which determine the entire equation. In regards to a particular kind of foam, at least seven experimental runs need to be carried out to predict the shrinkage ratio. Seven representative moulding conditions were selected randomly, and afterwards, the shrinkage ratios, $K_{cond}$ and $K_{rad}$, can be calculated in terms of moulding parameters. Finally, each coefficient value could be determined by using the Levenberg-Marquardt method of nonlinear least squares optimized algorithm. In this way, the shrinkage ratio equations of Foams I and II are shown as follows:

(19) $$SR=(6.6247-\frac{0.0525}{\sqrt{K_{cond}^{3}}}+8772.8232K_{rad})\cdot e^{\left(0.5165+\frac{0.0938}{\sqrt{K_{cond}}}+\frac{7.6488}{\ln K_{rad}}\right)}+0.0969$$

(20) $$SR=(18.4608-\frac{0.0128}{\sqrt{K_{cond}^{3}}}+2767.0344K_{rad})\cdot e^{\left(0.2234+\frac{0.0547}{\sqrt{K_{cond}}}+\frac{4.8058}{\ln K_{rad}}\right)}+0.1241$$

Both equations pass the significance test with decision coefficients by 0.87 and 0.93, respectively.

### 3.5. Verification of the Shrinkage Ratio Model

The 16 random verification runs were conducted by an experimental process. There were also three replicates of the thickness measurements for each group. In Table 3, the two rightmost columns are $SR_{\exp}$ and $SR_{pred}$, which indicate the experimental and predictive results of the shrinkage ratio, respectively. The remaining left columns are run number, foam type, and test conditions, respectively. The detailed moulding conditions include temperature, dwell time, and gap. $K_{cond}$ and $K_{rad}$ can be further calculated from Equations (9) and (10).

**Figure 4:**
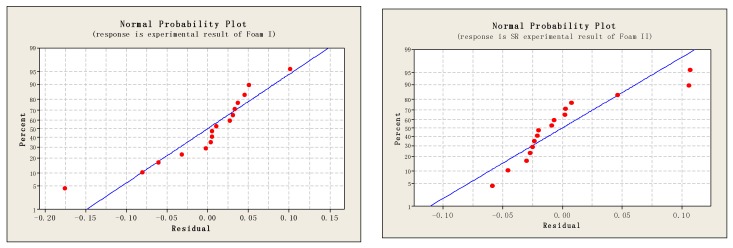
Residual analysis of normal probability plots for shrinkage ratio of Foams I and II.

The residual analysis of the normal probability plot indicates that the shrinkage ratio model is well available for both the fitting and the forecasting (see Figure 4). It provides a reliable and effective prediction of the post-moulding thickness of foam cups.

**Figure 5:**
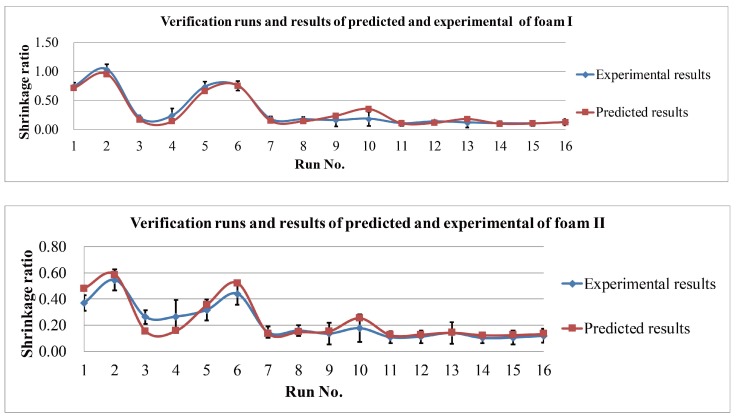
Simulation and experimental results of Foams I and II.

A comparison between the predictions and the measured data of the shrinkage ratio results from the experiments is shown in Figure 5. There is at least a 93.1% agreement in 16 different experimental conditions for both foam types between the experimental and simulation curves in these experiments. This might indicate that a higher order regression model for the control factors may not be required.

## 4. Conclusions

In this study, the heat transfer mechanisms in PU foam are discussed, and heat transfer modes are used to build an analytical model to estimate the shrinkage ratio. As the surfaces of bra cup mould heads are not parallel, the distance between each gap in the male and female mould heads is not the same. The post-moulding thicknesses of bra cups in different positions can be derived from corresponding gap distances between mould heads based on the shrinkage ratio equation in terms of different moulding conditions. This study has characterized heat transfer mechanisms of various material types in the moulding behavior, and it has also identified the relationships between foam properties, moulding conditions, and the shrinkage ratio of foams. Through the Levenberg-Marquardt method of nonlinear least squares algorithm, a prediction model is regressed to predict the shrinkage in the thickness of flexible PU foam after the moulding process. The application of the model can shorten the product development cycle, reduce design costs, and avoid duplication errors. Engineers and designers in the moulding design and processing sectors would benefit through an improved accuracy of processing predictions by the incorporation and use of moulding temperature, dwell time, gap between the two slabs, and foam material properties, as well as thermal conductivity data.

## Figures and Tables

**Table 1:** Specifications and physical properties of the 2 polyurethane (PU) foam material samples.

Style	Density (kg/m^3^)	Cell count (cells per 25 mm)	Tensile strength at 8% strain (kPa)	Compression stress at 40% strain (kPa)	Hardness (°ShD)	Cell type
Test standard	ISO845-1988	AS2282.5-1999	ISO1798-1983	ISO3386/1-1986	ASTM D2240-05	
Foam I	45.07	44.30	4.80	4.27	43.98	open
Foam II	28.23	46.20	2.80	2.16	21.96	open

**Table 2:** Radiative and conductive properties and the two coefficients in the exponential equation.

Foam type	Gap (m)	Temp. (K)	A	B	$R^{2}$	$K_{cond}$ (W/mK)	$K_{gas}$ (W/mK)	$K_{rad}$ (W/mK)	$K_{eff}$ (W/mK)
I	0.007	463	4.136	0.020	0.935	0.006	0.030	0.013	0.048
I	0.007	473	2.718	0.019	0.952	0.006	0.031	0.014	0.050
I	0.007	483	0.570	0.010	0.958	0.006	0.031	0.015	0.052
I	0.005	463	3.466	0.021	0.909	0.008	0.030	0.008	0.046
I	0.005	473	1.251	0.015	0.822	0.008	0.031	0.008	0.047
I	0.005	483	0.330	0.007	0.987	0.008	0.031	0.009	0.048
I	0.003	463	1.364	0.017	0.615	0.014	0.030	0.004	0.047
I	0.003	473	0.199	0.004	0.655	0.014	0.031	0.004	0.048
I	0.003	483	0.173	0.003	0.931	0.014	0.031	0.004	0.049
I	0.001	463	0.123	0.001	0.828	0.041	0.030	0.001	0.071
I	0.001	473	0.113	0.001	0.730	0.041	0.031	0.001	0.072
I	0.001	483	0.140	0.002	0.921	0.041	0.031	0.001	0.073
II	0.007	463	2.466	0.017	0.918	0.004	0.030	0.016	0.049
II	0.007	473	1.029	0.013	0.901	0.004	0.031	0.017	0.051
II	0.007	483	0.819	0.012	0.964	0.004	0.031	0.018	0.053
II	0.005	463	1.965	0.018	0.874	0.005	0.030	0.009	0.044
II	0.005	473	0.736	0.012	0.908	0.005	0.031	0.010	0.046
II	0.005	483	0.393	0.008	0.881	0.005	0.031	0.011	0.047
II	0.003	463	0.594	0.011	0.638	0.009	0.030	0.004	0.043
II	0.003	473	0.203	0.004	0.789	0.009	0.031	0.005	0.044
II	0.003	483	0.149	0.003	0.702	0.009	0.031	0.005	0.045
II	0.001	463	0.146	0.002	0.620	0.026	0.030	0.001	0.056
II	0.001	473	0.112	0.001	0.857	0.026	0.031	0.001	0.057
II	0.001	483	0.134	0.002	0.869	0.026	0.031	0.001	0.058

**Table 3:** The 16 random verification runs of each foam.

Run No.	Type	$K_{cond}$ (W/mK)	$K_{rad}$ (W/mK)	Time (s)	Gap (m)	Temp. (K)	$SR_{\exp}$	$SR_{pred}$
1	Foam I	0.00585	0.01288	90	0.007	463	0.7415	0.7117
2		0.00585	0.01373	60	0.007	473	1.0343	0.9535
3		0.00585	0.01373	120	0.007	473	0.2031	0.1728
4		0.00585	0.01462	90	0.007	483	0.2383	0.1393
5		0.00819	0.00778	90	0.005	463	0.7363	0.6633
6		0.00819	0.00829	60	0.005	473	0.7502	0.7553
7		0.00819	0.00829	120	0.005	473	0.1794	0.1481
8		0.00819	0.00883	90	0.005	483	0.1737	0.1390
9		0.01366	0.00361	90	0.003	463	0.1579	0.2355
10		0.01366	0.00385	60	0.003	473	0.1829	0.3508
11		0.01366	0.00385	120	0.003	473	0.1057	0.1055
12		0.01366	0.00410	90	0.003	483	0.1356	0.1115
13		0.04097	0.00070	60	0.001	463	0.1178	0.1783
14		0.04097	0.00070	120	0.001	463	0.1041	0.0979
15		0.04097	0.00074	90	0.001	473	0.1029	0.1015
16		0.04097	0.00079	60	0.001	483	0.1230	0.1282
1	Foam II	0.00367	0.01550	90	0.007	463	0.3707	0.4821
2		0.00367	0.01653	60	0.007	473	0.5457	0.5872
3		0.00367	0.01653	120	0.007	473	0.2628	0.1554
4		0.00367	0.01760	90	0.007	483	0.2657	0.1577
5		0.00513	0.00936	90	0.005	463	0.3166	0.3541
6		0.00513	0.00998	60	0.005	473	0.4396	0.5227
7		0.00513	0.00998	120	0.005	473	0.1456	0.1381
8		0.00513	0.01062	90	0.005	483	0.1585	0.1480
9		0.00855	0.00435	90	0.003	463	0.1358	0.1537
10		0.00855	0.00464	60	0.003	473	0.1767	0.2539
11		0.00855	0.00464	120	0.003	473	0.1082	0.1252
12		0.00855	0.00494	90	0.003	483	0.1123	0.1290
13		0.02566	0.00084	60	0.001	463	0.1407	0.1434
14		0.02566	0.00084	120	0.001	463	0.1022	0.1241
15		0.02566	0.00089	90	0.001	473	0.1055	0.1245
16		0.02566	0.00095	60	0.001	483	0.1186	0.1337
